# Dyspepsia and Sudden Death

**Published:** 1858-06

**Authors:** 


// HALL'S JOURNAL OF HEALTH.

OUR LEGITIMATE SCOPE IS ALMOST BOUNDLESS: FOR WHATEVER BEGETS PLEASURABLE AND HARMLESS FEELINGS, PROMOTES HEALTH; AND WHATEVER INDUCES DISAGREEABLE SENSATIONS, ENGENDERS DISEASE.

*We aim to show how Disease may be avoided, and that it is best, when sickness comes, to take no Medicine without consulting an educated Physician.*

VOL. VI.] JUNE, 1858. [No. 6.

### DYSPEPSIA AND SUDDEN DEATH.

A GENTLEMAN was one day standing at a gate, in the suburbs of London, talking to his daughter, and suddenly dropped down dead.

Sometimes sugar is found at the bottom of a cup, when the tea or coffee has been drunk off. But suppose the tea had not been drunk, but allowed to stand, it would in time wholly disappear, leaving the sugar at the bottom of the cup. This may show the unsophisticated reader, that although the sugar was dissolved in the cup of tea, it was not destroyed; but after a time, will appear in its native state. These two facts put together, may give a valuable lesson and a useful warning.

What killed the gentleman, while engaged in so pleasant an occupation as talking to his married daughter, before a little cottage in the vicinity of great London? On examination, it was found that the immediate cause of death was an impacted mass in the intestines, weighing several ounces, and this mass was reported to be soda. He had been a dyspeptic, and finding that soda alleviated his discomforts, he had fallen into the habit of taking it; this habit grew upon him, until he was swallowing large quantities every day. This alkali he dissolved in water, and sometimes ate it in its solid state, as one would eat a lump of sugar, to be dissolved by the saliva; but it was soda still; and thus it served him. Those who use soda daily, in whatever form, may learn from this fact a useful lesson. It is a very common expedient with ladies, when experiencing any discomfort from acidity, or any other result of indigestion, to take a little soda or saleratus. A beautiful, white, clean-looking powder is soda and saleratus, so "*simple*" too! made by putting a little pure water on the ashes of wood or sea-weed. It is quite as "simple" as the people who use it.

A great ado has been made in the papers by a number of persons, who, like the simpleton, "don't know what they don't know," and begin at once to talk about Albumen, Gastric Juice, Pancreatic Secretions, Free Hydrochloric Acid, and Occult Alkaline Matters; meanwhile, lookers-on draw conclusions for and against, as suits their purse or prejudices. Dr. YANKEE, who has books to sell, makes out a striking case, to the end that saleratus kills off half the children, and is killing off all the grown people, by rotting out their teeth, thus preventing them from masticating their food, and laying the foundation for dyspepsia; while his neighbor, who has *Dietetic Saleratus* to sell, declares that it *prevents* Dyspepsia, and causes a lump of dough which weighs seven pounds, .o weigh eight pounds, by the time it is baked. Then Dr. DENTIST insists on having his say, which is, that saleratus put in flour is transmogrified by the time it gets into the stomach, and is no saleratus at all; and if it is not saleratus, and the teeth do rot out, how can it be said that saleratus injures the teeth?

Thus it is, that between the three doctors, the common people conclude that the chances of their being mistaken are about equal; and that as it is very convenient to use saleratus and soda, and cream of tartar, in various preparations for the table, they might as well have the benefit which may result from their employment, and "trust to luck," for any possible ill results. Thus it is, that the use of these medical materials in our cookery is increasing, and scarcely any thing that is now made out of flour or meal, of any description, is free from these ingredients. But here is the man who dropped down dead, while conversing with his daughter, in consequence of a lump of soda having formed in his body, from his having used it so freely for indigestion.

Let the reader take another view of the case, in the light of chemistry. Saleratus is made out of the ashes of wood with pure water, and is a vegetable alkali. Soda is made out of the ashes of sea-weed with pure water, and is a mineral alkali; some use one, some use the other, in their cooking—their essential qualities being the same. But one undisputed fact stares us in the face; the fluids in the stomach which dissolve the food, in order to have the substance, the nutriment extracted from it, are acidulated, that is, contain an acid; if the acid is separated from this fluid, the food is not dissolved at all, is not fitted for digestion, but is simply decomposed, and acetic fermentation takes place; that is, the contents of the stomach become sour, and generate wind, precisely as is done in a barrel of sweet cider, and the bung is forced out by the accumulated air. Sometimes this generated air is got rid of by belching, or otherwise; if not, it causes the most familiar of all aches, with other things too tedious to mention.

But if the acids are not separated from the liquid which promotes digestion, but are *neutralized*, the effect is the same, it has not power to digest the food, sourness takes place, impure blood is the result, and general ill health prevails. So that alkalies, by neutralizing the acid of the fluid which promotes digestion, cannot be otherwise than injurious, if taken before, or at meal time, simply because they diminish the stomach's power of digestion. So that if soda or saleratus is in the food in any form whatever, it impairs digestion; and whatever does that, injures the health—and whatever does that, causes the teeth to decay. And those who will use it, in spite of these statements, on the ground that they do not use much, and that a little "can't do any great harm, anyway," will do well to remember, every drunkard used the same argument, when he first began to drink; and the principles of the saleratus-eater and the liquor-drinker are the same—neither of them are capable of self-control, and their appetites are the god of their idolatry.

Let it be fully and plainly understood by all, that human health can never be maintained by the habitual internal use of any thing else than plain, nourishing food, and such drinks as satisfy the thirst in a natural way. In other words, the habitual use of any thing, water excepted, which does not contain wholesome nutriment, tends, in all cases and under all circumstances, to destroy both health and life.

